# Preparation of Dye Semiconductors via Coupling Polymerization Catalyzed by Two Catalysts and Application to Transistor

**DOI:** 10.3390/molecules29010071

**Published:** 2023-12-22

**Authors:** Shiwei Ren, Wenqing Zhang, Zhuoer Wang, Abderrahim Yassar, Jinyang Chen, Minfeng Zeng, Zhengran Yi

**Affiliations:** 1 Zhuhai-Fudan Research Institute of Innovation, Hengqin 519000, China; shiwei_ren@fudan.edu.cn; 2 Zhejiang Key Laboratory of Alternative Technologies for Fine Chemicals Process, Shaoxing University, Shaoxing 312000, China; zengmf@usx.edu.cn; 3 Key Laboratory of Organic Solids, Institute of Chemistry, Chinese Academy of Sciences, Beijing 100190, China; 4 Key Laboratory of Colloid and Interface Chemistry of Chemistry and Chemical Engineering, Shandong University, Jinan 250100, China; 5 LPICM, Ecole Polytechnique, CNRS, Institut Polytechnique de Paris, 91128 Palaiseau, France; abderrahim.yassar@polytechnique.edu

**Keywords:** organic dye semiconductors, energy bandgap, transistor, electron mobility, optoelectronic applications

## Abstract

Organic dye semiconductors have received increasing attention as the next generation of semiconductors, and one of their potential applications is as a core component of organic transistors. In this study, two novel diketopyrrolopyrrole (DPP) dye core-based materials were designed and separately prepared using Stille coupling reactions under different palladium catalyst conditions. The molecular weights and elemental compositions were tested to demonstrate that both catalysts could be used to successfully prepare materials of this structure, with the main differences being the weight-average molecular weight and the dispersion index. PDPP-2Py-2Tz I with a longer conjugation length exhibited better thermodynamic stability than the counterpart polymer PDPP-2Py-2Tz II. The intrinsic optical properties of the polymers were relatively similar, while the electrochemical tests showed small differences in their energy levels. The polymers obtained with different catalysts displayed similar and moderate electron mobility in transistor devices, while PDPP-2Py-2Tz I possessed a higher switching ratio. Our study provides a comparison of such dye materials under different catalytic conditions and also demonstrates the great potential of dye materials for optoelectronic applications.

## 1. Introduction

Organic electronic materials are now being used in a wide range of applications for displays in mobile devices, as well as for other applications, such as organic photovoltaics, organic sensors, organic light-emitting transistors, organic electrochemical transistors and organic thin film transistors (OTFTs) [1,2,3,4]. One of the most fundamental and critical parameters for device applications is the carrier mobility. Depending on the type of carrier transport, materials can be simply categorized as n-type materials, which transport electrons, and p-type materials, which are hole transporting [5,6]. The current research on n-type materials is more relevant, due to the fact that most of the investigated materials for electron transport exhibit low mobility, and their research is less advanced in comparison to the research on p-type materials [7,8]. Many of the challenges in terms of improving and optimizing these applications, including mobility, are material-related and require the exploration of different molecular structures to identify the most suitable candidates. The low diversity of optional acceptor types and structures in the field of n-type materials limits the richness of the material’s structural library [9]. A well-established approach towards improving electron mobility is to design and synthesize new molecular structures of materials. Conjugated polymers, for example, are often based on donor–acceptor repeating units [10,11]. The chemical modification of the acceptor or donor unit to introduce strong electron-withdrawing functional groups or electronegative atoms can contribute to the overall electron-withdrawing ability of the material. Similarly, reducing or even eliminating the content of the donor from the donor–acceptor structure to the acceptor–acceptor architecture facilitates the reduction of the electron-donating properties of the material.

**Figure 1:**
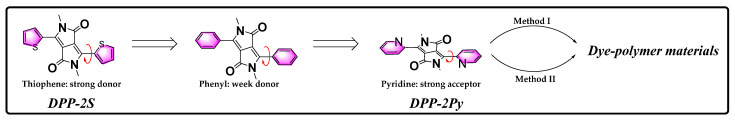
Molecular structure design strategies based on bridging motifs from donor to acceptor.

One of the most fundamental and common dye acceptor building blocks is based on the DPP, which is a conjugated structure with two carbonyl groups of a bicyclic ring [12,13]. The carbonyl group within the molecule exhibits strong electron-withdrawing properties and is the most widely used electron acceptor candidate. However, current research on DPPs is mainly based on DPP-2S, which is linked by two thiophenes on both sides of the DPP (Figure 1). From a synthetic point of view, the reasons for the interest in DPP-2S can be divided into two areas. The first is the ease of preparation of the product—DPP-2S can be prepared in high yields by the one-pot method when dicyanothiophene is available as a precursor; the second is the limited number of methods available for the straightforward preparation of DPPs that do not contain directly attached thiophene. The introduction of thiophene provides the advantage of producing a polymer based on the DPP-2S monomer, which can be polymerized by hydrocarbon activation or by the subsequent introduction of Br atoms in a Stille coupling polymerization reaction with Sn-containing monomers [14,15,16,17,18]. However, it has to be mentioned that thiophene acts as a strong donor, which is detrimental to the overall electron-withdrawing potency of the molecule. The n-type materials require a strong electron-withdrawing ability to ensure that the material possesses low highest occupied molecular orbital (HOMO) and low-lying lowest unoccupied molecular orbital (LUMO) energy levels, in order to guarantee the electron mobility of the device [19,20]. Therefore, the modification of DPP-2S might involve changing the strong donor of thiophene to a weak donor benzene ring [21]. Although the weak electron-donating ability of the benzene ring does not significantly affect the overall electron-withdrawing capacity of the material, its introduction brings an effect on the molecular planarity [22]. The torsion angle of the thiophene rings to the DPP ring is between 9° and 15°, while the benzene rings as the substituent show a torsion angle of roughly 40°. The reason for the large torsion angle is the close proximity between the hydrogen atoms on the benzene ring and the hydrogen atoms on the alkyl chain, which results in strong repulsive forces. Another key factor that determines the device mobility performance is the planarity of the molecular structure, as it determines the effective conjugation length on the molecular skeleton and the close stacking between molecules [23,24,25]. In order to further reduce the effect on the electron donor groups, as well as to further enhance the electron-withdrawing ability of the molecules, the introduction of pyridine rings is logical. The pyridine ring exhibits electron acceptor properties due to the presence of a strongly electronegative N atom [26,27]. On the other hand, the N in position 2 significantly weakens the space steric hindrance between the hydrogen atoms on the aromatic ring and the alkyl chain. Pop et al. point out that the orientation of thiophene is related to the type of alkyl chain, which leads, to some extent, to an increase in the number of isomers in the material [28]. In contrast, the pyridine ring favors the formation of an intramolecular conformational lock, thereby fixing the orientation of the pyridine ring with respect to the DPP. Therefore, the DPP with two pyridine rings as substituents, named DPP-2Py, should exhibit favorable coplanarity and a strong electron-withdrawing ability. Herein, we report a novel dye-polymeric material, PDPP-2Py-2Tz, based on an all-acceptor structure, which is based on a Stille coupling reaction to polymerize the monomer DPP-2Py with the monomer bithiazole (2Tz). The reason for the introduction of 2Tz is partly its strong electron-withdrawing properties and partly related to the good planarity presented by its molecule. The two thiazole units are connected by means of a single bond in a centrosymmetric manner, with a strong interaction between N···S to preserve their coplanar structure [29,30,31]. The objective of this research is to study the effect of different catalysts on the preparation and properties of the materials, such as thermal stability, LUMO energy levels, etc., and to investigate their impact on the performance of semiconductor devices.

## 2. Results

### 2.1. Synthesis Routes to Dye Polymer PDPP-2Py-2Tz via Two Different Stille Coupling Routes

One of the most common synthetic methods for donor–acceptor (D–A) polymers is the Stille coupling polycondensation reaction, which is also applicable to polymers based on the acceptor–acceptor (A–A) framework [32,33]. In this polymerization method, organo-distannane and organo-dihalogen compounds are treated as copolymerizing monomers to form long main chain polymers in the presence of a palladium catalyst. The other most frequently used method for the preparation of organic polymer semiconductor materials based on palladium metal catalysts is the Suzuki coupling reaction [34,35,36]. The best-known catalyst for both polymerization reactions is 3,3,6,6-tetramethyl-9-(1,2,3,4-tetrahydroxybutyl)-4,5,7,9-tetrahydro-2H-xanthene-1,8-dione, often named [Pd_2_(dba)_3_]. When a divalent palladium catalyst is used, it is often necessary to add additional reducing reagents, such as phosphorus ligands, to activate the catalyst’s activity. On the other hand, typical zero-valent palladium catalysts such as [Pd(PPh_3_)_4_] can generally be used directly and subjected to subsequent cycles of oxidative addition and reductive elimination processes to prepare the target product.

**Figure 2:**
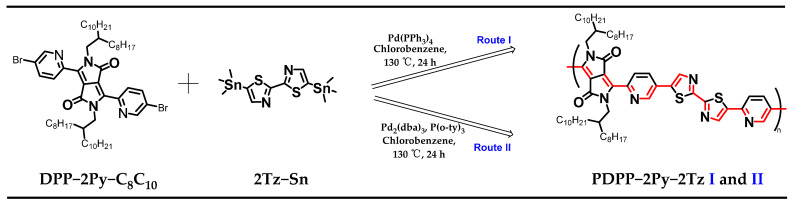
Synthesis process based on a palladium-catalyzed coupling polymerization reaction route, with alternating single and double bonds illustrated in red.

For DPP-2Py-based monomers, alkyl chains need to be introduced to improve the solubility and processability; therefore, the long chains are inserted here on both sides by an alkylation reaction. Another monomer selection procedure is based on the aim of promoting the full conjugation of the molecular architecture and low frontier orbital energy levels. The thiazole moiety possesses a strong electron-withdrawing capacity and rigid structural features. On the other hand, the enhancement of the polymers in long-range ordering is also related to the coplanarity of the comonomers composed, which contributes to the improvement of the material’s electron mobility [37]. Considering the effect of different catalysts on the molecular weight and optoelectronic properties of the final product, two types of palladium catalysts were investigated here, specifically [Pd_2_(dba)_3_] and [Pd(PPh_3_)_4_] [38]. The molar equivalence ratio of both catalysts was 4% relative to the monomer, so as to eliminate the potential effect of the amount of initiator on the molecular weight of the polymer during cross-coupling. In addition, the same solvents and polymerization times were selected to minimize the effect of condition factors on the conjugation length and molecular weight of the polymers. It is worth mentioning that the polymerization process of the two monomers took only 24 h, which distinguishes it from most cases, where the polymerization time reaches 3 days. The synthetic route and molecular structure of the polymer are shown in Figure 2, where each of its smallest repeating units bears an extremely long linear conjugated architecture with alternating single and double bonds, as shown in red. The purification of the dye polymers was carried out using the Soxhlet extraction technique. The recycling of hexane, acetone, methanol and ethyl acetate, respectively, effectively removes oligomers and short-chain polymers from the mixture. The lower molecular weight of the short-chain polymers rendered them soluble in non-chlorinated solvents, and they appeared as a blue-violet color, whereas the target products both appeared dark green in chloroform (Figure S1).

**Figure 3:**
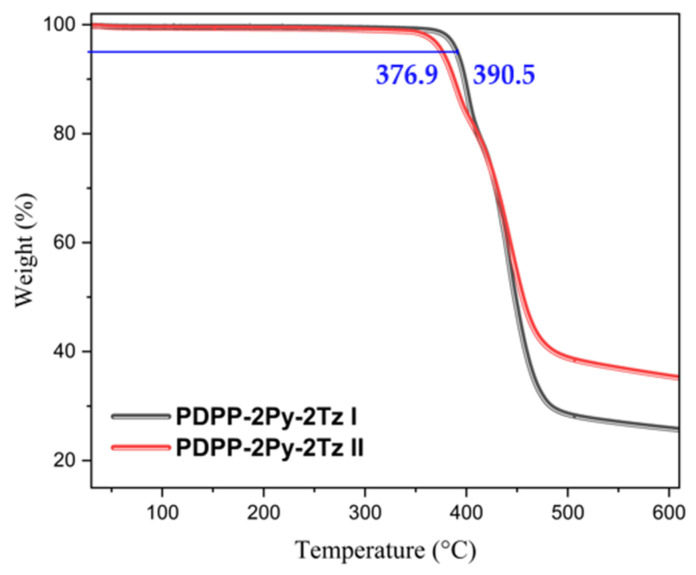
Comparison of the differences in the thermal decomposition curves of the polymers PDPP-2Py-2Tz I and PDPP-2Py-2Tz II and determination of the operating temperature of the materials during subsequent annealing.

The yields of the materials were comparable to those of most A–A-type polymers and slightly lower than those of preparations based on D–A-type materials. The yields of the two polymers were relatively comparable; however, their molecular weights showed a significant difference. The weight-average molecular weight (Mw) of the polymers synthesized according to Route I was 74.5 kDa, which was much higher than the value of the material synthesized by Route II, which was only 59.7 kDa (Table 1). The number of repeating units of polymers PDPP-2Py-2Tz I and PDPP-2Py-2Tz II was estimated to be 21 and 19, respectively, based on the number-average molecular weights (Mn), suggesting that the materials synthesized by route I exhibited longer conjugation lengths. On the other hand, the polymer dispersity index (PDI) obtained based on the ratio of Mw to Mn was 3.5 and 3.0, respectively, indicating that the homogeneity of the material obtained by the second method was slightly better. We speculate that this may be related to the size of the ligands contained in the different catalysts and thus the different efficiencies of the rate-determining step. Their specified values for the peak molecular weight and Z-mean molecular weight are shown in Figure S2. Based on the test results of the organic element analyzer, the C, H, N and S percentages of the material were 72.75%, 8.54%, 8.02% and 5.26%, respectively, which was quite close to the theoretical ratio of the smallest repeating unit, and it indicated that the materials were of a high degree of purity (Table 1). The precise elemental composition also indicates that the products include no catalyst or ligand components, which is necessary for the subsequent characterization of device properties. The infrared spectra of the two dye polymers were not obviously different, and the characteristic peaks of the carbonyl and alkyl chains were close to each other in terms of their peak-out positions (Figure S3). Chlorinated solvents were used in the subsequent characterization and measurements to maximize the possible solubility of the materials. Thermogravimetric analysis (TGA) was used to investigate the thermal properties of the polymers. As shown in Figure 3, both polymers were thermally stable, with decomposition temperatures higher than 350 °C, allowing film crystallinity and device performance analyses to be performed in the 150–300 °C annealing temperature range. Specifically, the two polymers exhibited different decomposition temperatures at 5% weight loss, which were 390.5 °C and 376.9 °C, respectively. This difference is significantly closer to 14 °C, which we believe is related to the variation in the molecular weights of the polymers under the prerequisite of the same structural composition.

### 2.2. Density Functional Theory (DFT) Calculation

**Figure 4:**
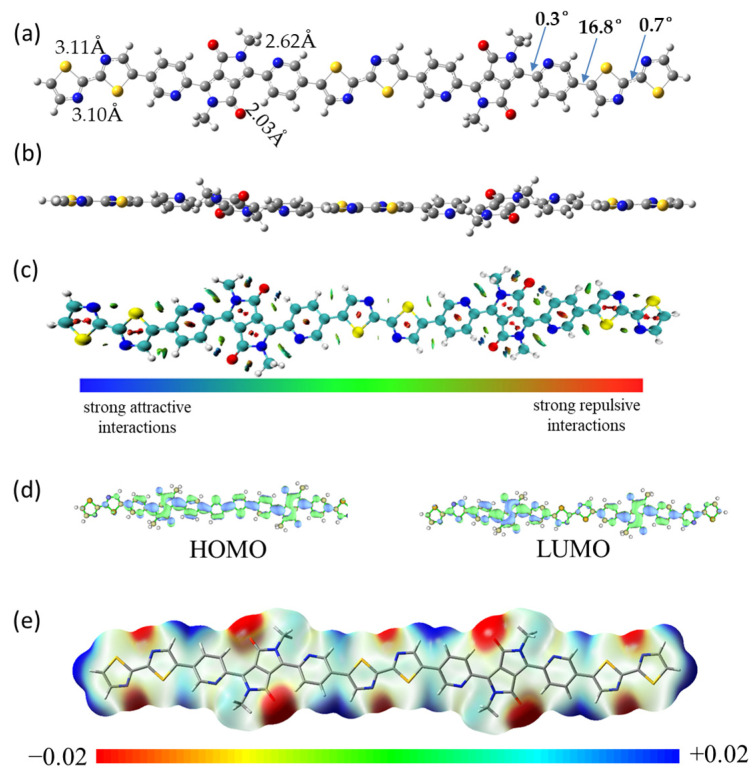
(**a**) Top view and (**b**) side view; (**c**) non-covalent interactions; (**d**) HOMO and LUMO map; (**e**) electrostatic potential surface of the optimized conjugated backbone conformation of PDPP-2Py-2Tz (methyl-substituted dimer).

We did not successfully complete the crystal growth of monomer DPP-2Py-C_8_C_10_ due to the length of the attached alkyl chains. Therefore, the measurement and characterization of the intramolecular structures of monomers and polymers are mainly based on theoretical simulations. The calculations demonstrate that the pyridine rings do not rotate freely, despite the fact that they are linked by means of a single bond. The close proximity of the nitrogen atom in the pyridine ring to the oxygen atom in the carbonyl group leads to strong repulsion and potential spatial hindrance. The most stable conformation is achieved when the dihedral angle is 0.3°, which is caused by the formation of attractive forces between the nitrogen atom and the hydrogen atom on the alkyl chain and between the hydrogen atom in the pyridine ring and the oxygen atom, respectively. The distance of O···H is 2.03 Å, which contributes to the formation of intramolecular hydrogen bonds and thus maintains the conformation of the system (Figure 4a). Hence, the monomer adopts the conformation with this lowest energy arrangement. A side view of the molecule, as shown in Figure 4b, demonstrates the planar regularity of its backbone. An analysis of the forces acting on some of the weak non-covalent bonds within the molecule is shown in Figure 4c, with the colors appearing blue and green [39]. Hydrogen bonding between the carbonyl oxygen and the hydrogen atoms on the pyridine ring is strong and appears in a blue color in their midst. The forces between the nitrogen atom on the pyridine ring and the hydrogen atom on the neighboring alkyl chain are relatively weak.

The model taken for the simulation of the polymer is a dimer and the long alkyl chains are replaced with methyl groups, which is associated with a reduction in the computational cost and time [40,41]. The dimer material as a whole exhibits a planar structure, which is favorable for the direct and hopping transport of electrons within and between the chains. The dithiazole exhibits a centrosymmetric conformation with a dihedral angle of 0.7°. The distances between the S and N atoms are 3.11 Å and 3.10 Å, respectively. The structure, which relies on a single bond connection, cannot be rotated arbitrarily. The dihedral angle between the pyridine ring and its directly bonded thiazole ring is 16.8°. Figure 4d shows the HOMO and LUMO energy level orbital maps of the materials with values of −5.52 eV and −3.42 eV, respectively. The energy gap obtained based on the difference between the HOMO and LUMO energy levels is 2.10 eV. The values for HOMO−1 and LUMO+1 are −5.61 eV and −3.42 eV, respectively (Figure S4). Figure 4e shows the charge distribution of the material, and it can be seen that there is a negative charge around the carbonyl group. All the above tests are based on the b3lyp-D3/def2tzvp unit of Gauss 16 [42,43,44,45,46]. Figure S5 shows the results of simulated UV with maximum absorption peaks at 434 nm and 639 nm, respectively. The onset of the absorption peak is 750 nm, which corresponds to a bandgap of 1.65 eV. The molar absorption coefficient and oscillator strength are 196 k cm^−1^M^−1^ and 2.1, respectively, revealing its good linear conjugation characteristics.

### 2.3. Photochemical Properties and Electrochemical Properties

**Figure 5:**
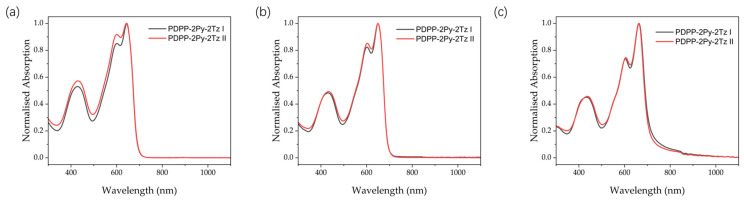
UV–vis absorption spectra of PDPP-2Py-2Tz (**a**) in chloroform solution; (**b**) in chlorobenzene solution; (**c**) in thin film.

The UV–Vis absorption spectra of the dye polymers in solution and in the solid state (thin films) are shown in Figure 5 below, and the corresponding data are collected in Table 2. The polymers, as with most of the reported polymers, show a dual absorption band. The high energy absorption band at 350−500 nm and the low energy absorption band at 500−700 nm correspond to π−π* transition and intramolecular charge transfer, respectively. Figure 5a illustrates the characteristic UV absorption patterns of the two dyes when chloroform is utilized as a solvent, with the main absorption peaks at 432 nm and 644 nm, respectively, accompanied by a shoulder peak at 600 nm. The change in the absorption peaks from chloroform to chlorobenzene is less obvious, and the only change is the red-shift of the maximum absorption (λ_max_) peak to 650 nm (Figure 5b). We believe that this slight redshift is related to the good solubility of the material in both solvents, and that its stacking in chlorobenzene may be more intense. The absorption bands of the two materials exhibit almost identical positions for the same solvent, with only the ratio of the 0–0 peaks to the 0–1 peaks showing a slight difference, which may be related to the homogeneity and dispersion of the materials. On the other hand, as shown in Figure 5c, the absorption in the solid state shows a significant variation, with its maximum absorption peak appearing at 665 nm, and the 15 nm bathochromic shift indicates that the material exhibits better intermolecular stacking in the solid state. The bandgap (E_g_^uv^) values of the polymers obtained based on measurements in the solution state are all in the vicinity of 1.77 eV, which is calculated based on the ratio of 1240 to the onset absorption peak (λ_onset_). The bandgap of the two polymers in the solid state is slightly different at 1.69 eV and 1.72 eV, respectively. It is worth mentioning that the test results of the actual UV absorption are in excellent agreement with the theoretical simulation diagram, and the minor inaccuracy mainly originates from the difference in the conjugation lengths between dimers and long-chain polymers.

**Figure 6:**
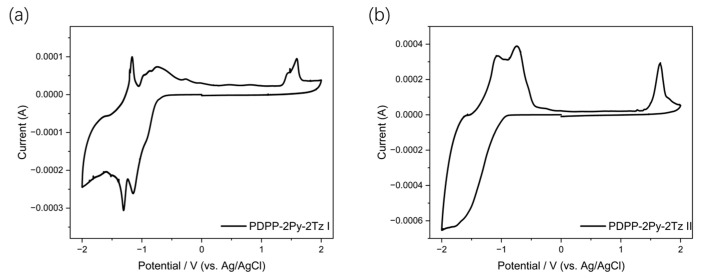
Redox curves of (**a**) PDPP-2Py-2Tz I; (**b**) PDPP-2Py-2Tz II (positive scan with the speed of 0.1 V/s).

The oxidation and reduction properties of the two polymers were characterized based on cyclic voltammetry and the results are shown in Figure 6. The overall reduction peaks of both materials are significantly larger than their oxidation peaks, which indicates the better potential of the materials for electron transport compared to hole transport. Based on the onset oxidation (E_ox_ ^onset^) and reduction (E_red_ ^onset^) peaks, the HOMO and LUMO energy levels of PDPP-2Py-2Tz I are obtained as −5.74 eV and −3.70 eV, while the HOMO and LUMO energy levels of PDPP-2Py-2Tz II are −5.82 eV and −3.60 eV, respectively. The small difference in LUMO energy levels may be related to the regularity of the molecule’s arrangement. Considering that the structural compositions of the polymers are almost the same, it is assumed that this difference in energy levels is mainly caused by the conjugation length of the polymers, i.e., the longer the conjugation length of the polymers, the smaller the energy level gap. Analogous to the energy levels obtained from the photochemical tests, there is a slight discrepancy in the energy level difference of the materials obtained from electrochemical analysis.

### 2.4. OTFT Performance

**Figure 7:**
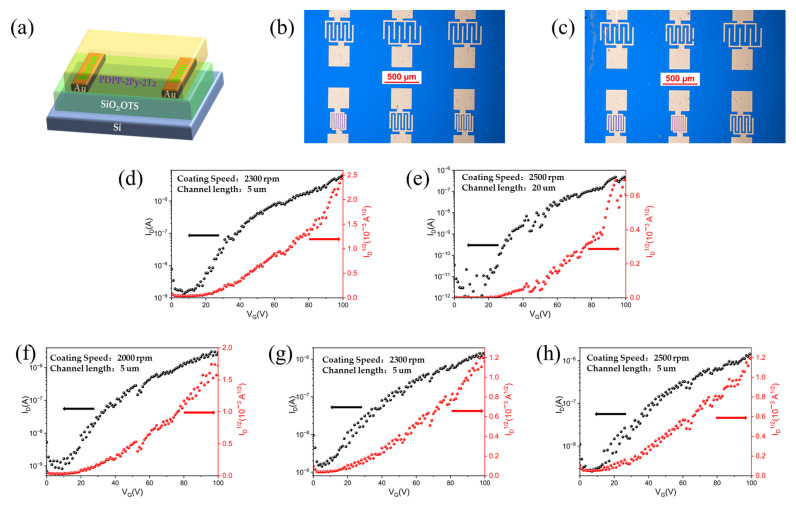
(**a**) OTFT devices with BGBC architectures; (**b**,**c**) optical microscope images of the OTFT devices; (**d**,**e**) transport characteristics of OTFT based on PDPP-2Py-2Tz I at different spin-coating speeds; (**f**–**h**) transport characteristics of OTFT based on PDPP-2Py-2Tz II at different spin-coating speeds.

OTFT devices with bottom gate–bottom contact (BGBC) architectures are exploited to characterize the carrier transport behavior of the materials (Figure 7a). Figure 7b,c show optical microscopy images of OTFT devices based on the two dye polymers with different channel lengths. Two types of materials based on polymer layers with thicknesses of approximately 20 to 50 nm were employed to investigate the conditions under which optimal performance emerges. Table 3 summarizes the electron mobility extracted from the transfer characteristic curve. As can be seen from Figure 7d–h, the PDPP-2Py-2Tz-based OTFT device possesses n-type electron unipolar transport properties. Both materials show moderate electron mobility, and the best performance is observed when the spin-coating speed is 2300 mm/s and 2000 mm/s for PDPP-2Py-2Tz I and PDPP-2Py-2Tz II, respectively. It is worth mentioning that the maximum current on/off ratio (I_ON_/I_OFF_) of 4.08 × 10^5^ was achieved in PDPP-2Py-2Tz I when the spin-coating speed was 2500 rpm, indicating its excellent switching property.

### 2.5. Thin Film X-ray Diffraction and Morphology

**Figure 8:**
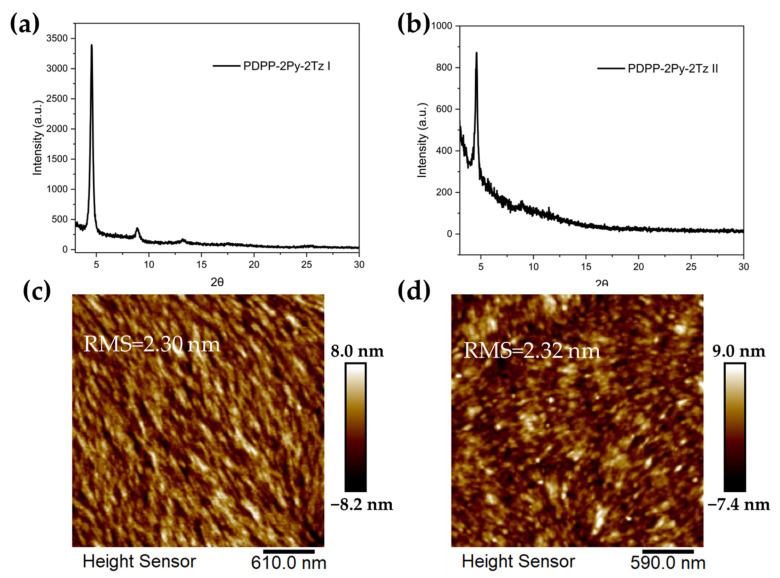
(**a**,**b**) Thin film XRD of PDPP-2Py-2Tz I and PDPP-2Py-2Tz II; (**c**,**d**) AFM height image of annealed films (220 °C) of PDPP-2Py-2Tz I and PDPP-2Py-2Tz II.

The molecular packing of the polymers was investigated by X-ray diffraction (XRD) experiments, which showed that the dye polymers were slightly crystalline after annealing (Figure 8a,b). There was an intense and sharp peak at 4.56° and 4.58° for PDPP-2Py-2Tz I and PDPP-2Py-2Tz II, respectively, indicating interchain d spacing of 19.37 Å and 19.30 Å. In contrast to PDPP-2Py-2Tz II, the low peak of PDPP-2Py-2Tz I near 24° (interlamellar d spacing of 3.71 Å) indicates that the latter is slightly more crystalline. We also employed an atomic force microscopy (AFM) measurement to investigate the surface morphologies of the thin films. As displayed in Figure 8c–d, both of the films exhibited smooth surfaces, with relatively small root-mean-square (RMS) roughness values of 2.30 and 2.32 nm for PDPP-2Py-2Tz I and PDPP-2Py-2Tz II, respectively, indicating the satisfactory solubility of the polymers before spin coating. It is noteworthy that both of the films exhibit fibrillar structures and appropriate phase separation sizes, which are beneficial for charge transportation.

## 3. Materials and Methods

Materials: The reaction precursor 3,6-bis(5-bromopyridin-2-yl)pyrrolo [3,4-c]pyrrole-1,4(2H,5H)-dione, named DPP-2Py, and the monomer 5,5′-bis(trimethylstannyl)-2,2′-bithiazole, named 2Tz-Sn, were purchased from SunaTech (Suzhou, China). The catalyst Tetrakis (triphenylphosphine) palladium [Pd(PPh_3_)_4_], bis(dibenzylideneacetone)palladium [Pd_2_(dba)_3_] and the organic solvents used, such as dimethylformamide, chlorobenzene, n-hexane, acetone, etc., were obtained from Merck without further purification. DPP-2Py-C_8_C_10_ was synthesized with reference to the previous literature and after optimizing the conditions in a 60% yield [47]. The monomers were characterized by nuclear magnetic resonance (NMR) spectra and their ^1^H NMR and ^13^C NMR are provided in Figures S6–S8 of the Supplementary Information (SI), respectively. ^1^H NMR (400 MHz, Chloroform-d) δ 8.93 (d, J = 8.6 Hz, 2H), 8.74 (s, 2H), 8.01 (dd, J = 8.6, 2.4 Hz, 2H), 4.28 (d, J = 7.3 Hz, 2H), 1.67–1.57 (m, 4H), 1.43–1.06 (m, 64H), 0.87 (td, J = 7.2, 6.8, 4.7 Hz, 12H). ^13^C NMR (100 MHz, Chloroform-d) δ 162.51, 150.12, 146.11, 144.99, 139.71, 128.48, 122.59, 111.46, 46.33, 38.24, 31.94, 31.91, 31.48, 30.02, 29.69, 29.67, 29.62, 29.56, 29.37, 29.33, 26.40, 22.70, 22.68, 14.11. Mass for C_56_H_88_Br_2_N_4_O_2_^+^: 1006.5274; Found: 1006.5276. 

Approach to the synthesis of PDPP-2Py-2Tz dye polymers: DPP-2Py-C_8_C_10_ (150.00 mg, 149.02 µmol, 1.0 eq), 2Tz-Sn (73.90 mg, 149.02 µmol, 1.0 eq) and tris(o-methylphenyl)phosphine (P(o-ty)_3_, 3.62 mg, 11.92 µmol) were dissolved in dry chlorobenzene (11 mL). The Schlenk tube was bubbled with argon for 15 min to eliminate oxygen from the system, followed by the rapid addition of catalyst ([Pd_2_(dba)_3_], 5.46 mg, 5.96 µmol, 4%). The polymerization was carried out with stirring at 130 °C for 24 h, with the completion of the polymerization process evidenced by a change in the color of the mixture to dark green and then a gradual return to room temperature. An alternative route to the synthesis of the polymer used the same chemical equivalent ratios and masses of monomers as the first, and the same ratios of chlorobenzene solvents. The main difference was that the catalyst type was changed to [Pd(PPh_3_)_4_] and no additional ligands were included. The purification process of both the prepared polymers was carried out by Soxhlet extraction, i.e., the short-chain oligomers were extracted with n-hexane, methanol, ethyl acetate and acetone, respectively, under heating and refluxing conditions, for 10 h each. The target polymers with high molecular weights were finally extracted, using chloroform as the extractant. The fractions were then evaporated and concentrated, precipitated into 150 mL of methanol and pump-filtered to afford the dark green powdery solid, which was dried under a vacuum for 7 h (85 °C) to obtain the polymeric material in 85% and 88% yields, respectively. The ^1^H NMR spectra of the polymers are provided in Figures S9 and S10.

**Figure 9:**
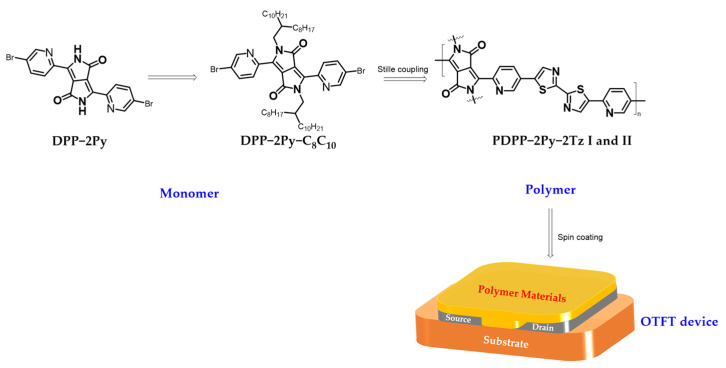
Schematic of the process from material to device.

OTFT-device: The OTFT was manufactured on heavily doped silicon (Si) wafers containing a silicon dioxide (SiO_2_) insulator as the gate. Gold (Au) electrodes were selected as the source and drain and fabricated via photolithography (30 nm). Ultrasound cleaning in deionized water, ethanol and isopropanol was carried out for 5 min each before the substrate was blow-dried using nitrogen gas. The channel width/length of the field effect transistor devices were 1400/5 and 1400/20 µm, respectively. The SiO_2_ gate dielectrics were treated with octadecyl-trichlorosilane (OTS) in a vacuum. The polymer was pre-dissolved in chlorobenzene at a high temperature (10 mg/mL, 80 °C) and then applied dropwise to the substrate by spin coating at 80 °C (2000−2500 rpm). The corresponding films were annealed at 220 °C for the removal of solvents (30 min). A brief schematic of the preparation of the materials and the application of the devices is shown in Figure 9 below.

Characterization: The NMR spectra of the organic molecules were determined on a Bruker AVANCE (400 MHz, Burker, Karlsruhe, Germany) instrument. Mass analyses were performed in ESI mode (SolariX, Karlsruhe, Germany). Elemental analysis was measured by the CHNS organic analyzer (FlashSmart, Thermo, Waltham, MA, USA). Infrared absorption peaks were analyzed by the Nicolet iS50 infrared spectrometer (Thermo, Waltham, MA, USA). The molecular weight of the PDPP-2Py-2Tz was assessed by gel permeation chromatography (150 °C, PL-GPC 220, Agilen, CA, USA). Trichlorobenzene was used for the eluent, and polystyrene was used as the standard. Thermal stability measurements were performed in the temperature range 30–600 °C and with a heating rate of 10 °C min^−1^ under a nitrogen environment (Rigaku, Tokyo, Japan). The photochemical test was performed on a UV–visible spectrometer (SPECTRONIC 300, Thermo, USA). Electrochemical profiling characterization was performed based on the electrochemical workstation (CHI440B, Shanghai, China) using cyclic voltammetry. The measurements were performed in a solution of acetonitrile with 0.1 M tetra-n-butylammonium hexafluorophosphate, and a three-electrode system was operated. The Ag/AgCl electrode, the glassy carbon electrode and the platinum electrode assumed the roles of the reference, working and counter electrodes, respectively. Then, 4 µL of polymer solution was pipetted into the working electrode dropwise and left to evaporate gradually. Optical microscope images of the OTFT devices were collected with an ECLIPSE LV100ND optical microscope (Nikon, Japan). The electrical performance of the OTFT device was assessed with a 4200-SCS semiconductor parameter analyzer in a nitrogen glovebox (Keithley, Cleveland, OH, USA). The mobility (*µ*) was calculated as follows: *µ* = ($\frac{\partial \surd {|I}_{DS}|}{\partial V_{GS}}$)^2^ ∙ $\frac{2L}{WCi}$. I_DS_: source–drain current; V_GS_: gate voltage; W: channel width; L: channel length; Ci: gate capacitance per unit area. Powder X-ray diffraction data were collected by acquiring thin films on Si/SiO_2_ wafers using a Bruker D8 Advance (Germany) Cu source diffractometer (40 mA, 40 KV). The scanning range was set from 0.5° to 30°, and the scanning speed was 1°/min. AFM measurements were performed on a Bruker Dimension ICON instrument (Mannheim, Germany). The samples were treated in exactly the same manner as those employed for the OTFT analyses.

## 4. Conclusions

In conclusion, a dye semiconductor, PDPP-2Py-2Tz, was prepared in this study by two methods. The [Pd(PPh_3_)_4_]-based polymerization was more efficient and resulted in polymeric materials with longer conjugation lengths at the same chemical equivalent catalyst usage. The thermal stability of the materials was further enhanced by increasing the molecular weight and conjugation length. The different main chain lengths marginally affected the energy levels of the materials. On the other hand, both materials exhibited moderate electron mobility due to their excellent conformation of coplanarity and the low-lying LUMO energy levels. The performance and device properties are not directly related to the choice of catalyst, and our study further illustrates the potential of [Pd(PPh_3_)_4_] as a catalyst for dye polymers with an acceptor–acceptor typology. Research on the electrical properties and optoelectronic applications of such dye-based materials is still ongoing in our lab.

## Figures and Tables

**Table 1:** Analytical results of the molecular weights of the two polymers and the ratio of the contained elements.

	Mn	Mw	PDI	C ^1^	H ^1^	N ^1^	S ^1^
				(%)	(%)	(%)	(%)
PDPP-2Py-2Tz I	21375	74488	3.48	72.75	8.54	8.02	5.26
PDPP−2Py−2Tz II	19876	59707	3.00	71.77	8.56	7.93	5.67
repeating unit	N/A	N/A	N/A	72.83	8.96	8.49	6.48

^1^ The average value of two tests.

**Table 2:** Main absorption peaks and energy level data.

	λ_max_^1^	λ_onset_^1^	E_g_^uv^	HOMO ^2^	LUMO ^3^	E_g_^cv^
	(nm)	(nm)	(eV)	(eV)	(eV)	(eV)
PDPP-2Py-2Tz I	665	734	1.69	−5.74	−3.70	2.04
PDPP−2Py−2Tz II	665	723	1.72	−5.82	−3.60	2.22

^1^ Solid-state results based on thin films; ^2^ E_HOMO_ = –4.8 − (E_ox_ ^onset^ – 0.40) eV; ^3^ E_LUMO_ = –4.8 − (E_red_ ^onset^ – 0.40) eV.

**Table 3:** Electron transport properties of PDPP−2Py-2Tz-based OTFT devices.

	Coating Speed (rpm)	Layer Thickness (nm) ^1^	Electron Mobility ^1^ (cm^2^/(V s)) × 10^−4^	I_ON_/I_OFF_
PDPP-2Py-2Tz I	2300	43	2.87	4.47 × 10^3^
PDPP-2Py-2Tz I	2500	34	0.63	4.08 × 10^5^
PDPP-2Py-2Tz II	2000	39	3.02	3.81 × 10^3^
PDPP-2Py-2Tz II	2300	30	1.03	1.07 × 10^3^
PDPP-2Py-2Tz II	2500	22	1.25	6.19 × 10^2^

^1^ Data are approximate values obtained using a Bruker DektakXT stylus surface profiler.

## Data Availability

Data are contained within the Supplementary Materials and the article.

## Supplementary Materials

The following supporting information can be downloaded at https://www.mdpi.com/article/10.3390/molecules29010071/s1: Figure S1: (a) Polymers PDPP-2Py-2Tz I (right) and PDPP-2Py-2Tz II (left) dissolved in chlorobenzene at room temperature and (b) at 80 °C; Figure S2: GPC data (cumulative percent curves and molecular weight distribution) for the two polymers; Figure S3: Infrared spectra of polymers PDPP-2Py-2Tz I (left) and PDPP-2Py-2Tz II (right); Figure S4: HOMO−1 and LUMO+1 map of PDPP-2Py-2Tz dimer; Figure S5: Simulated UV–vis spectra of PDPP-2Py-2Tz dimer; Figure S6: ^1^H NMR spectra of monomer 2FT-Sn; Figure S7: ^1^H NMR spectra of monomer DPP-2Py-C_8_C_10_; Figure S8: ^13^C NMR spectra of DPP-2Py-C_8_C_10_; Figure S9: ^1^H NMR spectra of PDPP-2Py-2Tz I; Figure S10: ^1^H NMR spectra of PDPP-2Py-2Tz II.
