# Supplementary material for: Preparation of Dye Semiconductors via Coupling Polymerization Catalyzed by Two Catalysts and Application to Transistor
Source: Molecules. 2023 Dec 22;29(1):71. doi: 10.3390/molecules29010071 (PMC10780007; doi:10.3390/molecules29010071)
Supplement: Supplementary file 1 [file molecules-29-00071-s001.zip › molecules-2767773-supplementary.pdf]

Supplementary Information

Figure S1. (**a**) Polymers PDPP-2Py-2Tz I (right) and PDPP-2Py-2Tz II (left) dissolved in chlorobenzene at room temperature and (**b**) at 80 °C.

(a) (b)

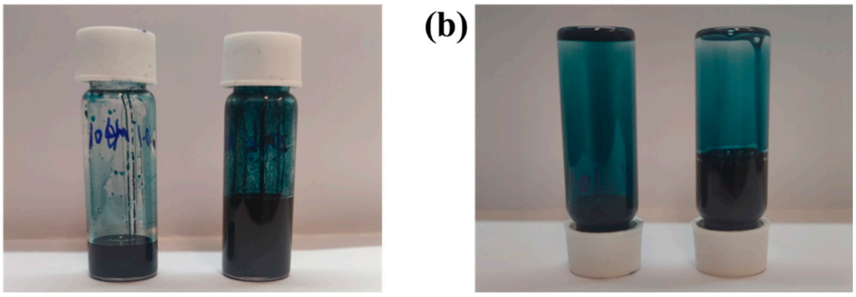

There are small solid particles precipitated at room temperature. Completely dissolved when heated up to 80 °C.

Figure S2. GPC data (cumulative percent curves and molecular weight distribution) for the two polymers.

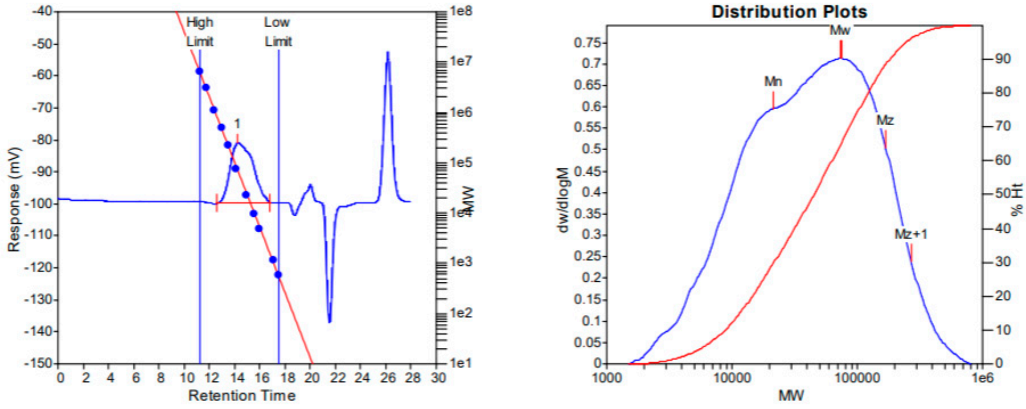

MW Averages

Peak No	Mp	Mn	Mw	Mz	Mz+1	Mv	PD
1	73083	21375	74488	169743	271206	62639	3.48482

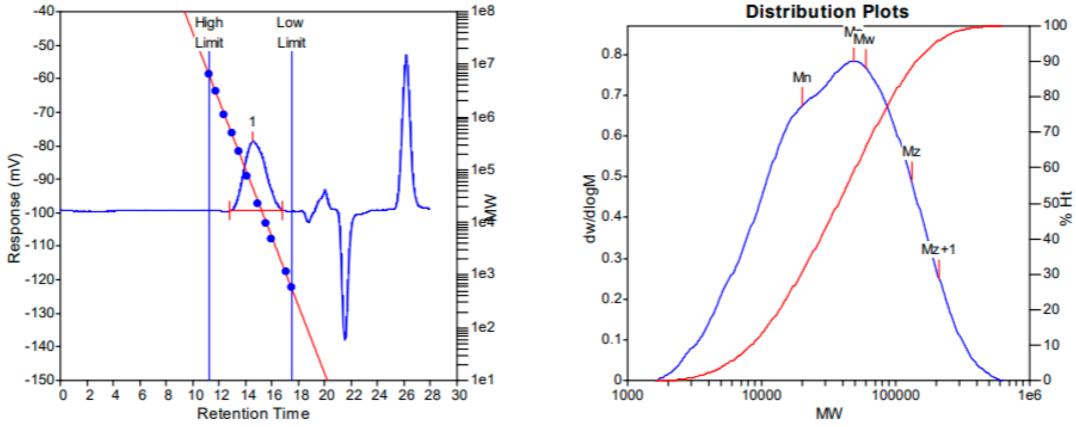

MW Averages

Peak No	Mp	Mn	Mw	Mz	Mz+1	Mv	PD
1	49079	19876	59707	131582	210425	50881	3.00397

Processed Peaks

Peak No	Name	Start RT (mins)	Max RT (mins)	End RT (mins)	Pk Height (mV)	% Height	Area (mV.secs)	% Area
1		12.80	14.50	16.78	20.8084	100	2454.71	100

Figure S3. Infrared spectra of polymers PDPP-2Py-2Tz I (left) and PDPP-2Py-2Tz II (right).

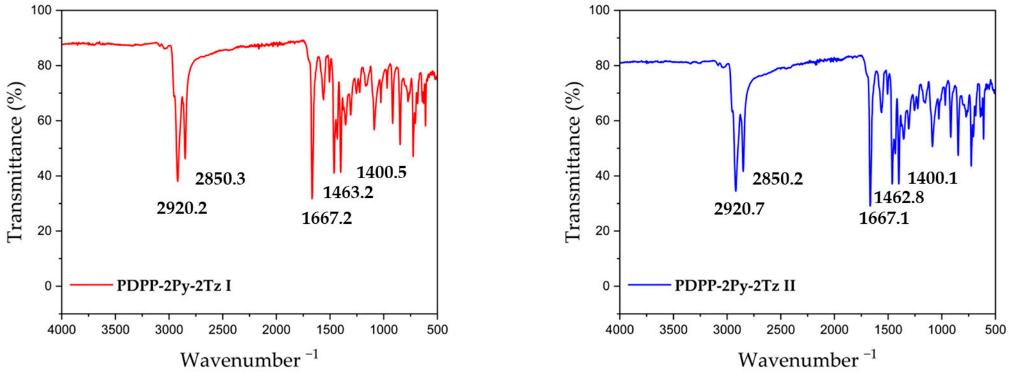

Figure S4. HOMO–1 and LUMO+1 map of PDPP-2Py-2Tz dimer.

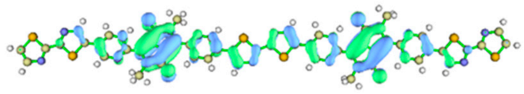

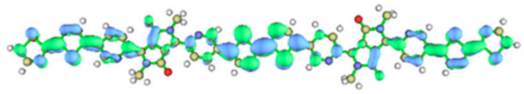

Figure S5. Simulated UV-vis spectra PDPP-2Py-2Tz dimer

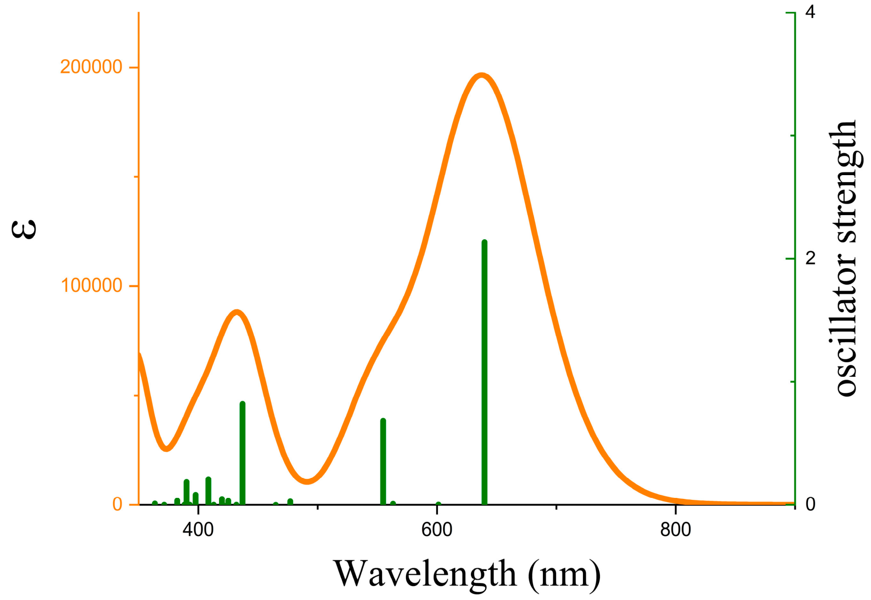

Figure S6. ^{1}H NMR spectra of monomer 2Tz-Sn

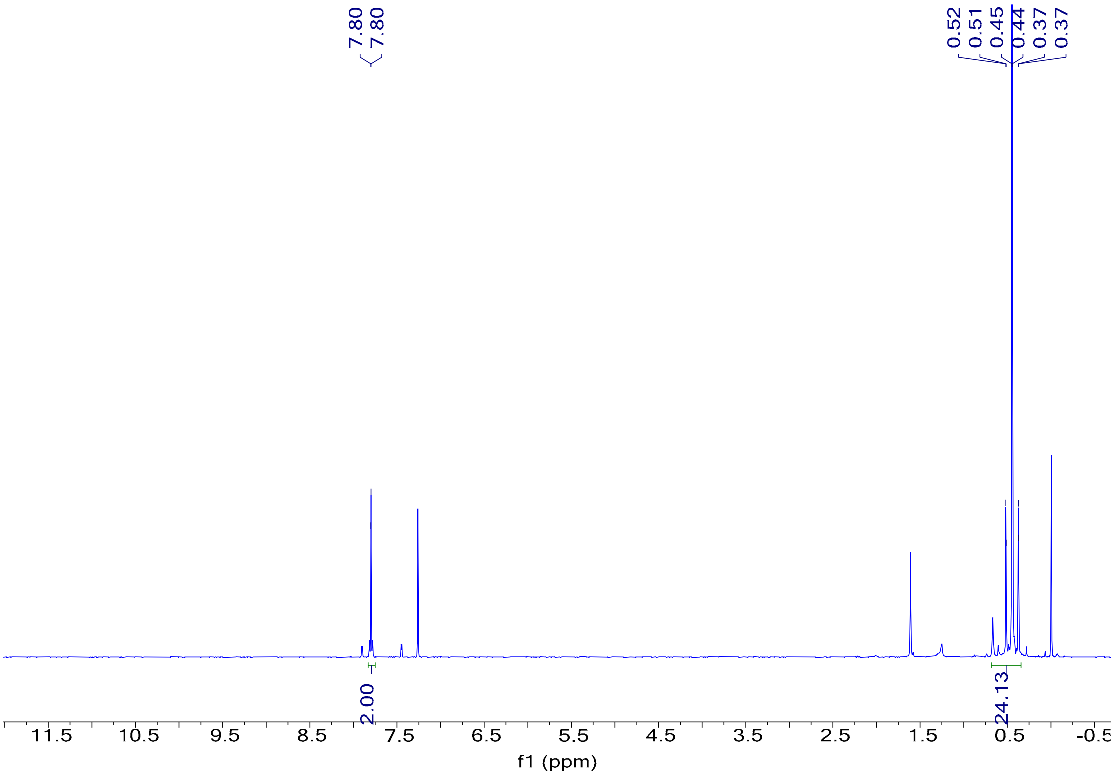

Figure S7. ^{1}H NMR spectra of monomer DPP-2Py-C_8C_{10}

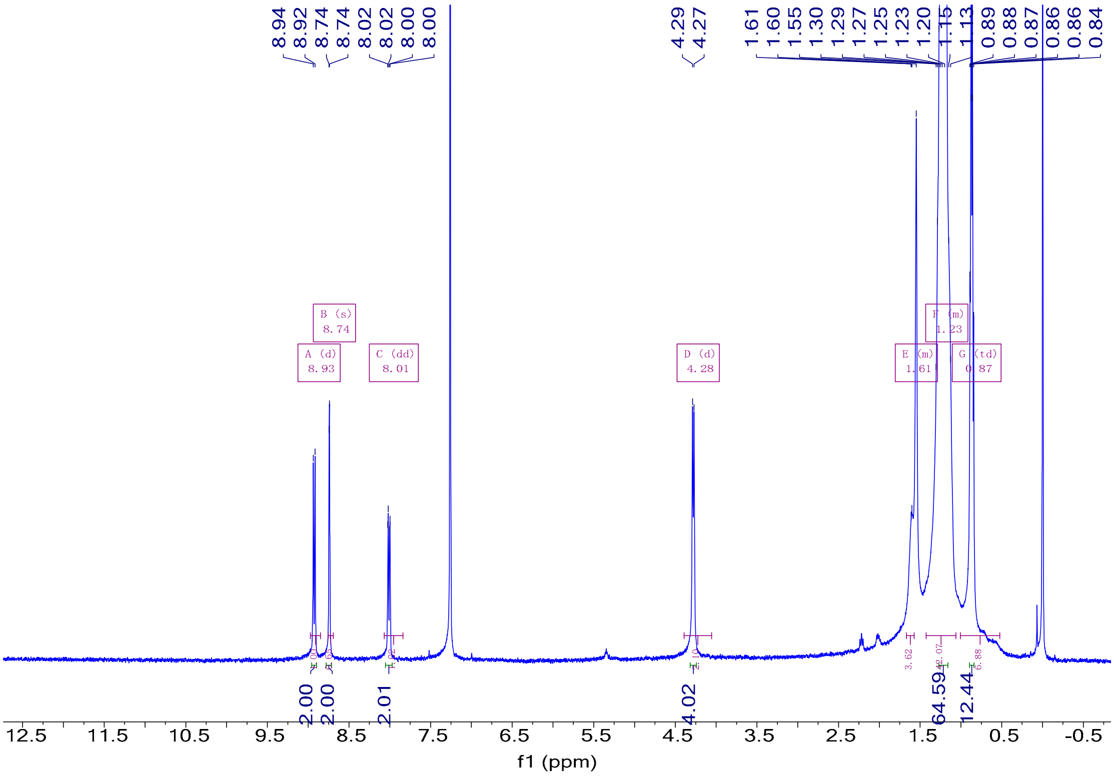

Figure S8. ^{13}C NMR spectra of monomer DPP-2Py-C_8C_{10}

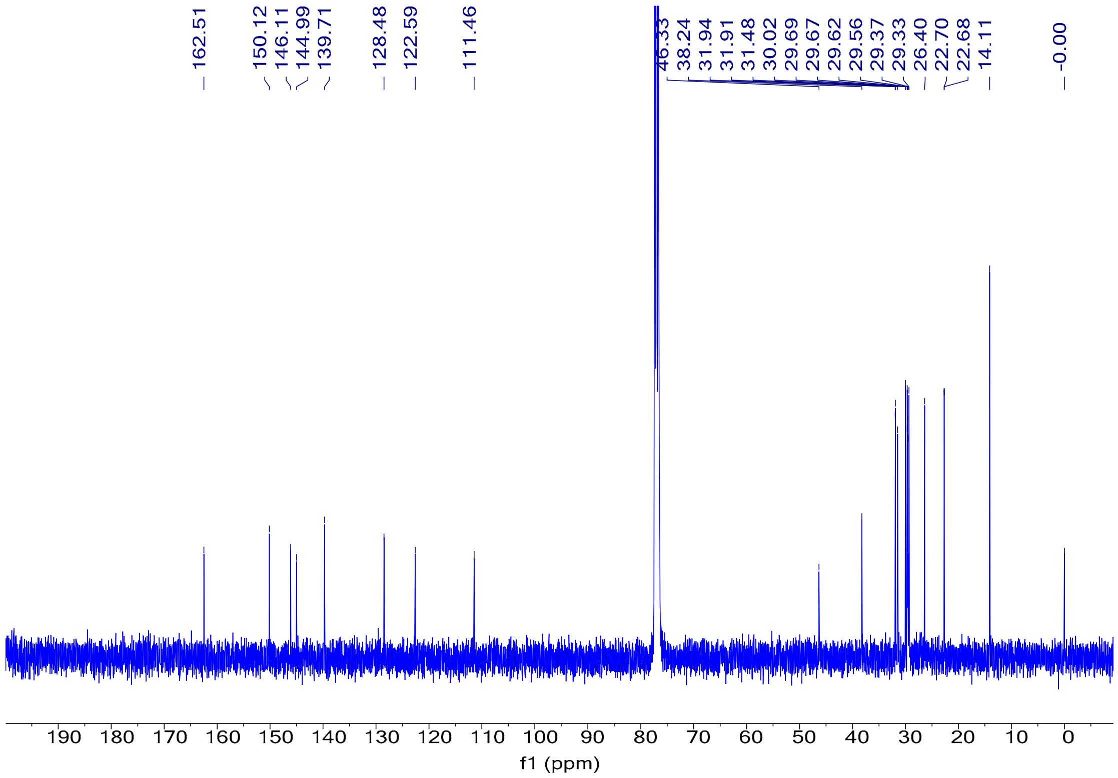

Figure S9. ^{1}H NMR spectra of PDPP-2Py-2Tz I

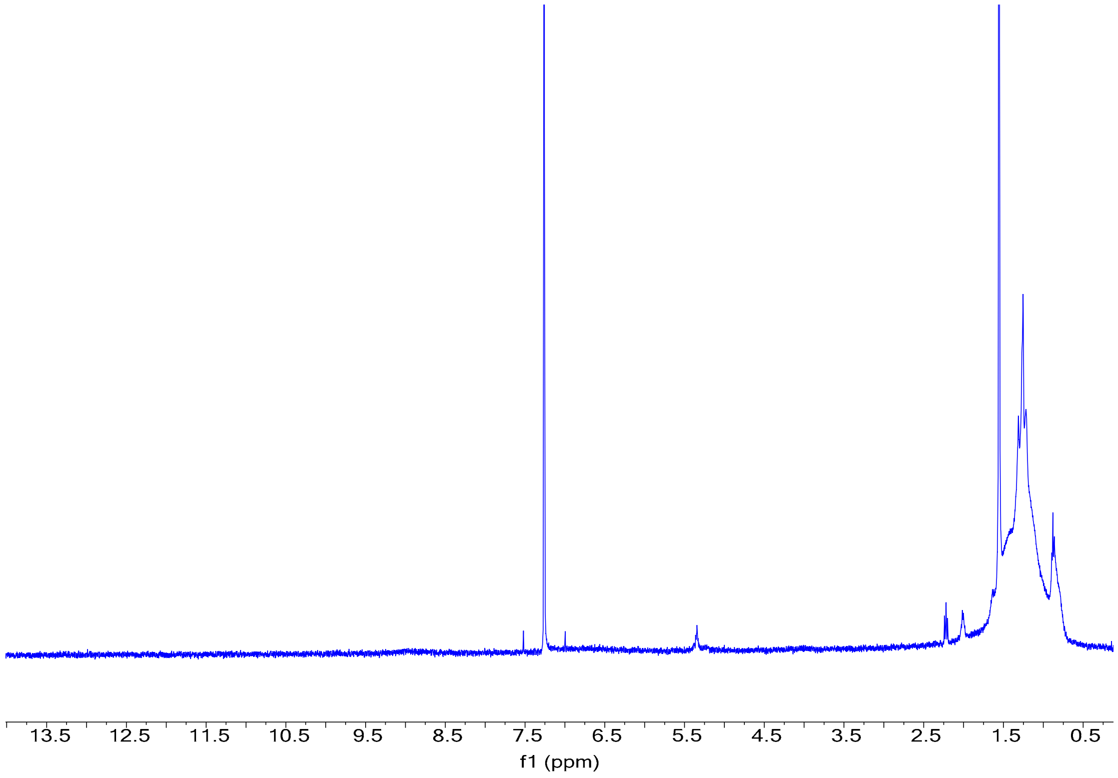

Figure S10. ^{1}H NMR spectra of PDPP-2Py-2Tz II

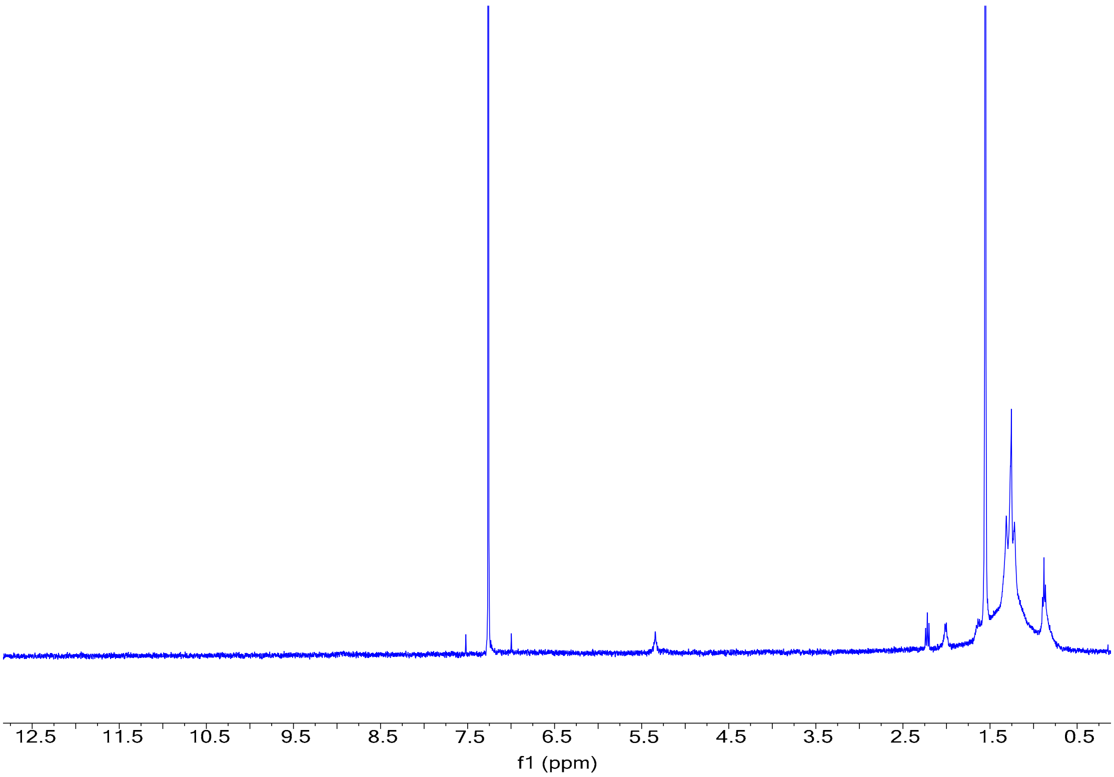

E = –5535.250915 Hartree, Imaginary frequency = 0;

C	8.71297400	-0.56190300	-0.27278000
C	9.74932900	0.30898800	0.03993700
C	10.98897500	-0.36251400	-0.12328000
C	10.70744400	-1.71688700	-0.56040500
N	9.28240000	-1.77261000	-0.63193000
C	10.03109700	1.66299300	0.47882900
N	11.45582300	1.71781300	0.55332100
C	12.02527000	0.50740400	0.19346500
O	11.42071500	-2.66802000	-0.83377000
O	9.31768500	2.61464000	0.74988600
C	13.45476500	0.24182100	0.16785100
C	7.28337200	-0.29732700	-0.24233000
C	8.63826000	-3.00818500	-1.04924600
C	12.10020200	2.95470400	0.96667500
N	14.29829200	1.22630400	0.52107800
C	15.59754500	0.99484500	0.50183200
C	16.17729700	-0.22688600	0.12558100
C	15.28995400	-1.24486700	-0.24619500
C	13.93055300	-1.02101100	-0.22238800
N	6.43845500	-1.28853500	-0.57298800
C	5.13910700	-1.05826700	-0.54830700

C	4.56095600	0.16909800	-0.18905300
C	5.44946200	1.19440900	0.15949700
C	6.80878000	0.97143100	0.13035000
C	17.61503400	-0.42591200	0.12753900
C	3.12328600	0.36723100	-0.18540500
S	18.75274100	0.88636400	0.17156700
C	20.05058500	-0.26975000	0.15611800
N	19.66404800	-1.51901400	0.11968700
C	18.31135000	-1.61386300	0.10301100
C	21.42901900	0.15456600	0.18019600
N	21.81033300	1.40422000	0.22327600
C	23.16960500	1.50156000	0.23513900
C	23.84207900	0.31341700	0.20082900
S	22.73431300	-0.99630400	0.15110600
S	1.98675700	-0.94584700	-0.18041800
C	0.68806300	0.21019300	-0.19160100
N	1.07492600	1.46118100	-0.19626700
C	2.42648300	1.55628000	-0.19205400
C	-0.68809200	-0.21040700	-0.19190100
N	-1.07495600	-1.46138900	-0.19776200
C	-2.42651500	-1.55649000	-0.19384900
S	-1.98679200	0.94562700	-0.17987500

C	-12.02526800	-0.50745300	0.19299000
C	-10.98899000	0.36231800	-0.12421100
C	-9.74934600	-0.30920200	0.03896600
C	-10.03110100	-1.66307100	0.47828300
N	-11.45581600	-1.71779500	0.55306300
C	-10.70746000	1.71663300	-0.56151300
N	-9.28241700	1.77232000	-0.63313800
C	-8.71299500	0.56164300	-0.27388300
O	-9.31768700	-2.61469100	0.74943000
O	-11.42072800	2.66774800	-0.83494600
C	-7.28339100	0.29707200	-0.24339200
C	-13.45475600	-0.24180200	0.16757200
C	-12.10018300	-2.95455800	0.96682100
C	-8.63829000	3.00773800	-1.05093400
N	-6.43845800	1.28839700	-0.57366200
C	-5.13910700	1.05814600	-0.54890700
C	-4.56098300	-0.16931700	-0.18995400
C	-5.44950000	-1.19474400	0.15821400
C	-6.80882100	-0.97178300	0.12897800
N	-14.29827200	-1.22619400	0.52108400
C	-15.59751900	-0.99468300	0.50199900
C	-16.17726800	0.22701700	0.12564700

C	-15.28994200	1.24490300	-0.24642000
C	-13.93054600	1.02098900	-0.22279000
C	-3.12331000	-0.36744400	-0.18623600
C	-17.61499800	0.42611200	0.12779500
S	-18.75275900	-0.88612000	0.17173000
C	-20.05054900	0.27005900	0.15662400
N	-19.66395800	1.51931200	0.12035800
C	-18.31125600	1.61409900	0.10354500
C	-21.42900200	-0.15419500	0.18078700
N	-21.81037000	-1.40383800	0.22368200
C	-23.16964600	-1.50111500	0.23568600
C	-23.84206700	-0.31293500	0.20167100
S	-22.73424400	0.99674400	0.15206000
H	9.44169300	-3.71334400	-1.24735700
H	7.98628500	-3.39155600	-0.26853700
H	8.04385700	-2.85456200	-1.94628400
H	12.68777200	2.80597800	1.86895000
H	12.75890600	3.33040600	0.18788700
H	11.29729200	3.66343500	1.15381000
H	16.23102600	1.82039300	0.81151000
H	15.66495900	-2.20632000	-0.56961600
H	13.23850300	-1.80147800	-0.50866100

H	4.50437200	-1.88950600	-0.83987400
H	5.07535100	2.16052400	0.46991800
H	7.50193300	1.75655800	0.40084400
H	17.85353200	-2.59185600	0.09184200
H	23.63274000	2.47645900	0.27007600
H	24.90657700	0.14888000	0.20271900
H	2.88504400	2.53371700	-0.21478700
H	-2.88509000	-2.53389800	-0.21756600
H	-11.29726800	-3.66325600	1.15406800
H	-12.68765500	-2.80556700	1.86911300
H	-12.75897500	-3.33046200	0.18820400
H	-7.98639200	3.39148400	-0.27034500
H	-8.04380700	2.85373600	-1.94785600
H	-9.44173200	3.71278000	-1.24942400
H	-4.50434700	1.88947400	-0.84016500
H	-5.07538600	-2.16092300	0.46843400
H	-7.50198900	-1.75697800	0.39923800
H	-16.23099400	-1.82015000	0.81190300
H	-15.66496500	2.20631900	-0.56993200
H	-13.23850500	1.80137400	-0.50930900
H	-17.85338400	2.59206900	0.09249500
H	-23.63282300	-2.47599900	0.27049800

H	-24.90655700	-0.14834700	0.20371500
